# Vesicular Stomatitis Virus Infection Promotes Immune Evasion by Preventing NKG2D-Ligand Surface Expression

**DOI:** 10.1371/journal.pone.0023023

**Published:** 2011-08-09

**Authors:** Helle Jensen, Lars Andresen, Jens Nielsen, Jan Pravsgaard Christensen, Søren Skov

**Affiliations:** 1 Laboratory of Immunology, Section of Biomedicine, LIFE, University of Copenhagen, Copenhagen, Denmark; 2 National Veterinary Institute, Technical University of Denmark, Lindholm, Denmark; 3 Institute of International Health, Immunology and Microbiology, University of Copenhagen, Copenhagen, Denmark; McMaster University, Canada

## Abstract

Vesicular stomatitis virus (VSV) has recently gained attention for its oncolytic ability in cancer treatment. Initially, we hypothesized that VSV infection could increase immune recognition of cancer cells through induction of the immune stimulatory NKG2D-ligands. Here we show that VSV infection leads to a robust induction of MICA mRNA expression, however the subsequent surface expression is potently hindered. Thus, VSV lines up with human cytomegalovirus (HCMV) and adenovirus, which actively subvert the immune system by negatively affecting NKG2D-ligand surface expression. VSV infection caused an active suppression of NKG2D-ligand surface expression, affecting both endogenous and histone deacetylase (HDAC)-inhibitor induced MICA, MICB and ULBP-2 expression. The classical immune escape mechanism of VSV (i.e., the M protein blockade of nucleocytoplasmic mRNA transport) was not involved, as the VSV mutant strain, VSV^ΔM51^, which possess a defective M protein, prevented MICA surface expression similarly to wild-type VSV. The VSV mediated down modulation of NKG2D-ligand expression did not involve apoptosis. Constitutive expression of MICA bypassed the escape mechanism, suggesting that VSV affect NKG2D-ligand expression at an early post-transcriptional level. Our results show that VSV possess an escape mechanism, which could affect the immune recognition of VSV infected cancer cells. This may also have implications for immune recognition of cancer cells after combined treatment with VSV and chemotherapeutic drugs.

## Introduction

Vesicular stomatitis virus (VSV) is an oncolytic virus that can replicate rapidly in a large number of tumor cell lines and selectively kill them. In addition, VSV exhibits a tumor repression effect *in vivo* in both human tumor xenografts in nude mice and syngenic tumors in immune competent mice [1], [2].

The RNA genome of VSV encodes five viral proteins (i.e. nucleoprotein, phosphoprotein, matrix (M) protein, glycoprotein and a large polymerase protein) [3]. The M protein is involved in transcriptional regulation [4] and in packaging and budding of virions [5], [6]. A primary function of the M protein is however to obstruct cellular antiviral programs. It can shut off host cell expression of antiviral gene products, such as type 1 interferons, by blocking the nucleocytoplasmic transport of host cell RNA [7], and this leads to an inhibition of host innate immune mechanisms [8]. The inhibition of mRNA export has been found to involve an interaction between the M protein and nuclear pore (i.e. Nup98) and export (i.e. Rae1) proteins [9], [10]. The mutant VSV strain, VSV^ΔM51^, harbor a defect M protein that is no longer able to inhibit the nucleocytoplasmic transport of RNA, nevertheless this virus retain its full oncolytic effect [8], [11].

The immune system needs to recognize and subsequently eliminate infected or transformed cells. A major surveillance mode is governed by the NKG2D/NKG2D-ligand system. Healthy human cells express low levels of NKG2D-ligands, whereas expression is up-regulated on bacterial/viral infected cells and on many tumors and otherwise stressed cells. Several different forms of NKG2D-ligands exist, primarily belonging to the *MIC* or *RAET1* (*ULBP*) gene families [12], [13]. The existence of different ligands for the same NKG2D receptor is puzzling and may reflect an evolutionary protection against pathogen mediated down modulation of particular NKG2D-ligands. The activating receptor, NKG2D, is expressed by several cells of the immune system, including human NK cells, CD8 T cells, some CD4 T cells, NK-T cells and γδ T cells, and ligation of the receptor leads to immune activation and an elimination of the NKG2D-ligand expressing cells [12], [14]–[16].

A number of viruses, including human cytomegalovirus (HCMV) and adenovirus, induce NKG2D-ligand expression [13], [17]. However, in order to escape immune recognition HCMV and adenovirus can hinder cell surface expression of some NKG2D-ligands. The HCMV UL16/UL142 protein can retain ULBP-1, ULBP-2, MICB and some alleles of MICA (i.e. it can only bind the full-length MICA alleles and not the truncated allele, MICA*008) in the endoplasmic reticulum (ER)/Golgi apparatus [18]–[22]. Likewise, MICA and MICB can be retained in the ER by the adenovirus E3/19K protein following infection [23].

Histone deacetylase (HDAC)-inhibitors is a new class of chemotherapeutic agents, which are able to increase surface expression of NKG2D-ligands in stressed and tumorigenic cells [24]–[26]. HDAC-inhibitors do not affect VSV infection in normal tissue, but potently enhance oncolytic VSV killing of cancer cells both *in vitro* and *in vivo* [27]. It is therefore possible that HDAC-inhibitors act as cancer specific VSV sensitizers.

This study shows that even though VSV infection leads to gene activation of the NKG2D-ligand, MICA, there is an inhibition of NKG2D-ligand surface expression early after transcription. Interestingly, this is not caused by a M protein mediated block of nucleocytoplasmic transport. Our results provide evidence that VSV possess an as yet unknown escape mechanism targeting the NKG2D/NKG2D-ligand surveillance system. Our results further suggest that care should be taken when VSV is used for cancer therapy, especially when the NKG2D/NKG2D-ligand surveillance system is involved.

## Materials and Methods

### Cell lines

Two Jurkat T cell lines were used in this study: JE6-1, from American Type Culture Collection, and JTag-9, kindly provided by Dr. Carsten Geisler (Department of International Health, Immunology and Microbiology, University of Copenhagen, Denmark). JTag-9 cells are stably transfected with the large T antigen from SV40 and they were only used for transient transfection studies. The melanoma cell lines, FM-86 and FM-78, were kindly provided by Dr. Per thor Straten (CCIT, Department of Hematology, Herlev University Hospital, Denmark). All the cells were grown in RPMI-1640 medium (Sigma-Aldrich) supplemented with 10% FBS, 2 mM glutamine, 2 mM penicillin and streptomycin.

### Reagents, plasmids and antibodies

FR901228 was obtained from the National Cancer Institute (Bethesda, MD). ZVAD-Fmk, propidium iodide (PI) and cycloheximide (CHX) was purchased from Sigma-Aldrich. IFN-α was kindly provided by Dr. Niels Ødum (Department of International Health, Immunology and Microbiology, University of Copenhagen, Denmark). The MICA*008-GFP vector construct was kindly provided by Dr. Mark R. Wills (Department of Medicine, University of Cambridge, U.K.) [18]. The Flag-Triad3A vector construct was kindly provided by Dr. John Hiscott (Department of Medicine, McGill University, Canada) [28]. To generate the MICA*009-GFP vector construct, RNA was isolated from Jurkat T-cells using TRIzol reagent (Invitrogen) and reverse-transcribed using oligo(dT)_20_ primers and the SuperScript III reverse transcriptase enzyme (Invitrogen). PCR was performed under standard conditions using a PfuUltra II DNA polymerase (Stratagene). MICA primer sequences were: MICA_cDNA_fwd: 5′-CACCATGGGGCTGGGCCCGGTCTTCCT-3′; and MICA_cDNA_nostop_rev: 5′-CGGCGCCCTCAGTGGAGCCAGTGGA-3′. The PCR product was cloned into pcDNA3.1/CT-GFP-TOPO (Invitrogen) using TOPO technology. The following antibodies were used for flow cytometry analysis: PE-conjugated MICA/B Ab (558352; BD Biosciences); APC-conjugated Annexin V (550474; BD Biosciences); PE-conjugated ICAM-1 Ab (C170; Leinco Technologies); PE-conjugated ULBP-2 Ab (FAB1298P; R&D Systems); APC-conjugated ULBP-2 Ab (FAB1298A; R&D Systems); PE-conjugated IgG Ab (555787; BD Biosciences) and APC-conjugated IgG Ab (555576; BD Biosciences).

### Viruses

VSV10 is a wild-type strain of the Indiana serotype. VSV^ΔM51^ was kindly provided by Dr. Chantal G. Lemay (University of Ottawa, Center for Cancer Therapeutics, Canada). VSV^ΔM51^ is a mutated strain of the Indiana serotype. It has a single amino acid deletion of methionine at position 51 of the matrix (M) protein and contains an extra cistron that encodes GFP, inserted between the sequences encoding the glycoprotein and large polymerase protein [8].

### Transient transfections

JTag-9 cells were transiently transfected with the Nucleofector kit V (Amaxa, Inc) according to the manufacturer's protocol. Briefly, 2×10^6^ cells were spun down, resuspended in 100 µl Cell Line Nucleofector Solution V, mixed with 5 µg vector construct and pulsed using the Nucleofector Program G10. For infection with VSV, the cells were rested for 2 hr before infected with 0.001 or 0.01 MOI VSV10 or VSV^ΔM51^ for 19 hr. For treatment with CHX, the cells were rested for 4 hr before treated with 10 µg/ml CHX for 15 hr or the cells were rested for 12 hr before treated with 20 µg/ml CHX for 7 hr.

### Flow cytometry analysis

Antibody surface staining of cells was done as previously described [25]. For surface staining with Annexin V, cells were washed and stained in buffer containing 10 mM Hepes (pH 7.4), 0.14 M NaCl, and 2.5 mM CaCl_2_. Intracellular staining with MICA/B Ab was done using the BD Cytofix/Cytoperm kit (BD Biosciences) according to the manufacturer's protocol. For staining with PI, cells were incubated for at least 5 min at RT with 1 µg/ml PI. Flow cytometry analysis and data acquisition were performed on a BD FACSCanto II or FACSCalibur. All results show forward-scatter on a linear scale and fluorescence on a log10 scale.

### Real-time RT-PCR analysis

For measurement of MICA mRNA level following VSV10 or VSV^ΔM51^ titration, 2×10^6^ JE6-1 cells were mock infected (with media) or infected with 0.001, 0.01 or 0.1 MOI of VSV10 or VSV^ΔM51^ for 19 hr and RNA was isolated using a MagNa Pure LC System (Roche Diagnostics) or TRIzol reagent (Invitrogen). For measurement of MICA mRNA level following time titration of FR901228 treatment or VSV10 infection, 3×10^6^ JE6-1 cells were treated with 20 ng/ml FR901228 or infected with 0.1 MOI VSV10 for 0, 4, 8, 12, 15 or 18 hr and RNA was isolated using TRIzol reagent. Real-time RT-PCR analysis of MICA mRNA expression was done as previously described [25]. The housekeeping gene Ribosomal Protein, Large, P0 was used as a loading control. The quantitative real-time PCR analysis was performed using the Brilliant SYBR Green QPCR Master Mix kit (Stratagene) and run on a Stratagene Mx3000P thermocycler.

### Sequencing of M protein in VSV^ΔM51^

JE6-1 cells were infected with 0.1 MOI VSV^ΔM51^ for 19 hr and RNA was extracted in a MagNa Pure LC System (Roche Diagnostics). The RNA was reverse-transcribed using random hexamer primers and the SuperScript III reverse transcriptase enzyme (Invitrogen). PCR was performed under standard conditions using a Taq DNA polymerase (New England BioLabs). Specific primers (VSV_M_fwd: 5′-ACGAATTCAAATTAGGGATCGCACCACC-3′ and VSV_M_rev: 5′-ACGGATCCCGTGATACTCGGGTTGACCT-3′) were used to amplify a 377-bp fragment spanning bases 61–438 of the VSV M gene. The PCR product was cloned into a pCR4-TOPO vector using the TOPO TA Cloning Kit for Sequencing (Invitrogen), which was subsequently used to transform One Shot chemically competent TOP10 cells (Invitrogen). From the selective plate several colonies were chosen for analysis and subcultured overnight at 37°C. Plasmid DNA was extracted from the cultures using a QIAprep Spin Miniprep Kit (QIAGEN GmbH), according to the manufacturer's protocol. The plasmid DNA was sequenced at MWG-Biotech (Germany).

### Statistics

When applicable, data are presented as mean±SD. For comparison of groups (treated versus non-treated samples) statistical analysis was performed using one-way ANOVA followed by Dunnett's test. Significance was defined as *p*<0.05.

## Results

### Surface expression of NKG2D ligands is down modulated upon VSV infection

**Figure 1:**
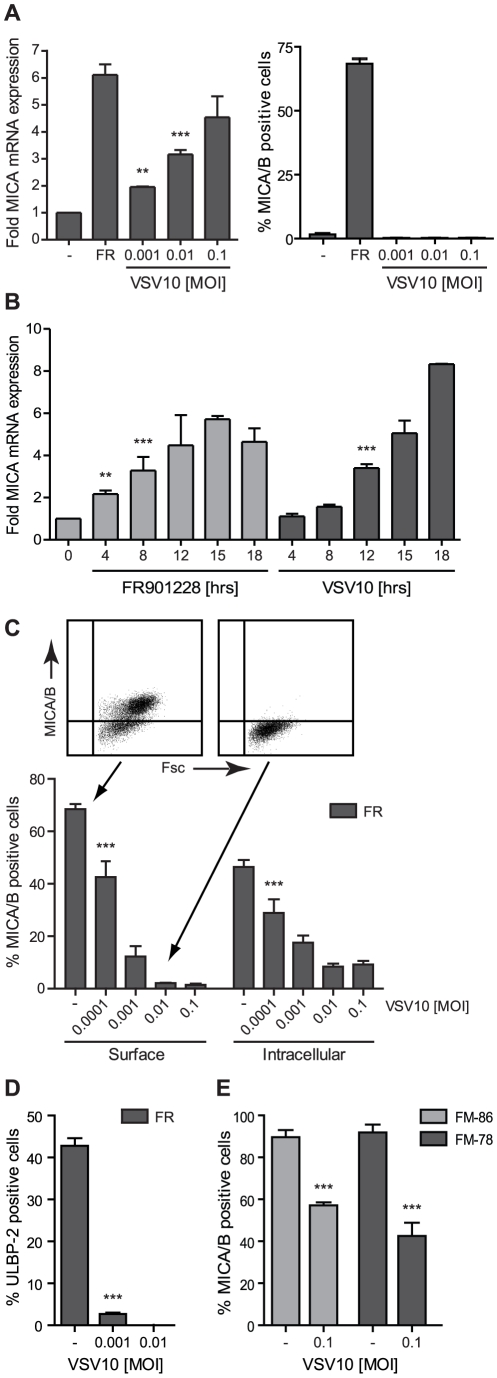
Surface expression of NKG2D ligands is down modulated upon VSV infection. ***A***, JE6-1 cells were either mock infected+non-treated with FR901228 (-), mock infected+treated with 20 ng/ml FR901228 for 18 hr (FR) or infected with the indicated MOI of VSV10 for 19 hr. The MICA mRNA level was examined by real-time RT-PCR and surface expression of MICA/B by flow cytometry. The MICA mRNA level is displayed as fold expression relative to the mock infected sample. The bar graphs show mean±SD from four experiments. ***B***, JE6-1 cells were either treated with 20 ng/ml FR901228 or infected with 0.1 MOI VSV10 for 0, 4, 8, 12, 15 or 18 hr. The MICA mRNA level was examined by real-time RT-PCR. The MICA mRNA level is displayed as fold expression relative to the mock treated/infected sample. The bar graphs show mean±SD from two experiments. ***C*** and ***D***, JE6-1 cells were mock infected (-) or infected with the indicated MOI of VSV10 one hr prior to treatment with 20 ng/mL FR901228. 19 hr post infection, the cells were analyzed for MICA/B or ULBP-2 surface expression or intracellular MICA/B expression by flow cytometry. The bar graphs show mean±SD from three experiments. ***E***, The melanoma cell lines, FM-86 and FM-78, were mock infected (-) or infected with 0.1 MOI VSV10 for 19 hr and analysed for MICA/B surface expression by flow cytometry. The bar graphs show mean±SD from two experiments. For all the bar graphs, ** p<0.05 and *** p<0.0001.

Jurkat T-cells were infected with VSV10 and examined for NKG2D-ligand expression. Interestingly, VSV10 infection leads to a robust induction of MICA mRNA expression (Fig. 1A), to a level resembling the induction by HDAC-inhibitor, FR901228, which is a well-known inducer of NKG2D-ligand expression [24], [25]. Combined VSV10 infection and FR901228 treatment led to a further increase in MICA mRNA expression (data not shown). An up-regulation of MICA mRNA expression in Jurkat T-cells could be observed 4 hr post treatment with FR901228, whereas an up-regulation of MICA mRNA following VSV10 infection was observed after 12 hr (Fig. 1B). Strikingly no surface expression of MICA was observed on the VSV10 infected cells (Fig. 1A). Moreover, the FR901228-mediated (Fig. 1C) or the endogenous (data not shown) MICA/B expression on Jurkat T-cells was significantly hampered by VSV10 infection. The down modulation of FR901228-induced MICA/B cell surface expression was detectable 12 hr post infection (data not shown). We also observed a significant down modulation of the structural different NKG2D-ligand, ULBP-2, following FR901228 treatment and VSV infection (Fig. 1D). In addition, VSV10 infection significantly down modulated the constitutive surface expression of MICA/B on the two melanoma cell lines, FM-86 and FM-78 (Fig. 1E). No inhibition of the surface protein ICAM-1 was observed on the Jurkat T-cells following VSV10 infection (data not shown).

These results show that VSV10 infection induce transcriptional activation of the stress induced NKG2D-ligand MICA, without subsequent MICA surface expression. This is most likely due to a post transcriptional down modulation of NKG2D-ligands, as VSV10 decreases the HDAC-inhibitor mediated and endogenous MICA surface expression, without affecting the mRNA level.

### Interferon-α restores FR901228-induced MICA/B surface expression after VSV infection

VSV is known to inhibit the nucleocytoplasmic transport of several host cell RNAs through its matrix (M) protein [9]–[11], which could be a likely explanation for the reduced NKG2D-ligand expression observed after VSV10 infection.

**Figure 2:**
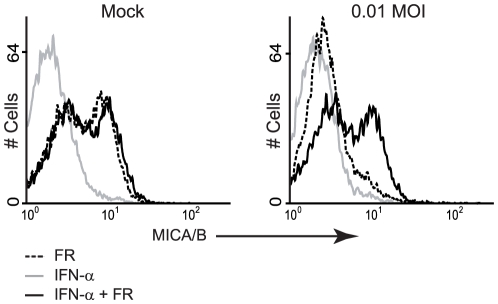
Interferon-α restores FR901228-induced MICA/B surface expression after VSV infection. JE6-1 cells were treated twice with 1000 U IFN-α with a 24 hr interval. One hr after the second treatment with IFN-α, the cells were mock infected or infected with 0.01 MOI VSV10. One hr post infection the cells were treated with 20 ng/mL FR901228 (FR), where indicated. 19 hr post infection, the cells were analyzed for MICA/B surface expression by flow cytometry. The data represent one out of two experiments.

Treatment of cells with IFN-α increases Nup98 expression, which subsequently reduces the ability of the M protein to inhibit nucleocytoplasmic transport of RNA [29]. Additionally, IFN-α alleviates several virus inflicted obstructions of cellular functions. We did not detect any MICA/B surface expression on VSV10 infected cells after pretreatment with IFN-α (Fig. 2). However, FR901228-mediated surface MICA/B expression was rescued by pretreatment with IFN-α (Fig. 2), suggesting that VSV and FR901228 induce MICA/B surface expression through different pathways. IFN-α is known to inhibit the infectious potential of VSV [8], which may also add to the results.

### Infection with the M protein mutated virus strain, VSV^ΔM51^, blocks NKG2D-ligand surface expression

**Figure 3:**
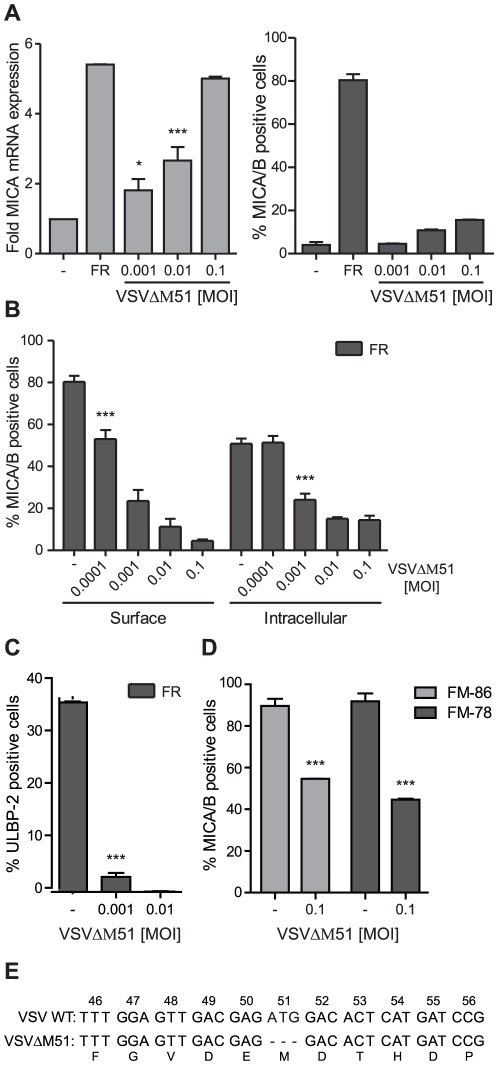
Infection with the M protein mutated virus strain, VSV^ΔM51^, blocks NKG2D-ligand surface expression. ***A***, JE6-1 cells were either mock infected+non-treated with FR901228 (-), mock infected+treated with 20 ng/ml FR901228 for 18 hr (FR) or infected with the indicated MOI of VSV^ΔM51^ for 19 hr. The MICA mRNA level was examined by real-time RT-PCR and surface expression of MICA/B by flow cytometry. The MICA mRNA level is displayed as fold expression relative to the mock infected sample. The bar graphs show mean±SD from four and three experiments, respectively. ***B*** and ***C***, JE6-1 cells were mock infected (-) or infected with the indicated MOI of VSV^ΔM51^ one hr prior to treatment with 20 ng/mL FR901228. 19 hr post infection, the cells were analyzed for MICA/B or ULBP-2 surface expression or intracellular MICA/B expression by flow cytometry. The bar graphs show mean±SD from three experiments. ***D***, The melanoma cell lines, FM-86 and FM-78, were mock infected (-) or infected with 0.1 MOI VSV^ΔM51^ for 19 hr and analysed for MICA/B surface expression by flow cytometry. The bar graphs show mean±SD from two experiments. ***E***, The VSV^ΔM51^ M protein was sequenced as described in section *2.7*. The VSV^ΔM51^ clone insert was aligned to the gene bank sequence of M protein (VSV WT; M11754.1; Indiana serotype of VSV). For all the bar graphs, * p<0.05 and *** p<0.0001.

The amino acid, methionine, at position 51 of the VSV M protein is essential for inhibition of the nucleocytoplasmic transport of host cell RNAs [8], [11]. Thus, it was interesting if the VSV^ΔM51^ strain, which has the methionine deleted from the M protein, was able to induce surface expression of NKG2D-ligands. A strong increase in the MICA mRNA level was observed after VSV^ΔM51^ infection (Fig. 3A) and as expected combined VSV^ΔM51^ infection and FR901228 treatment led to a further increase in MICA mRNA expression (data not shown). Unexpectedly, VSV^ΔM51^ infection did only result in a minor level of MICA/B surface expression (Fig. 3A). To substantiate this finding, we treated the cells with HDAC-inhibitors and found the same level of inhibition of ULBP-2 and MICA/B expression (Fig. 3B & C), as observed with the wild-type VSV10 strain. Additionally, VSV^ΔM51^ infection inhibited endogenous MICA/B and ULBP-2 surface expression on Jurkat T-cells (data not shown) and constitutive MICA/B surface expression on the two melanoma cell lines, FM-86 and FM-78 (Fig. 3D). ICAM-1 surface expression remained unchanged on Jurkat T-cells infected with VSV^ΔM51^ (data not shown).

It is interesting that VSV^ΔM51^, which is engineered not to inhibit nucleocytoplasmic transport of RNA, could still inhibit HDAC-inhibitor mediated and endogenous surface expression of NKG2D-ligands. To exclude the possibility that VSV^ΔM51^ had reverted to wild-type VSV (i.e. lost its mutation of the M protein), we sequenced the M protein of virus collected from infected Jurkat T-cells and confirmed the mutation (Fig. 3E).

These results indicate that VSV infection down modulates NKG2D-ligand surface expression by a novel mechanism, not dependent on M protein-mediated nucleocytoplasmic transport.

### VSV-mediated down modulation of MICA/B surface expression is not caused by apoptosis

**Figure 4:**
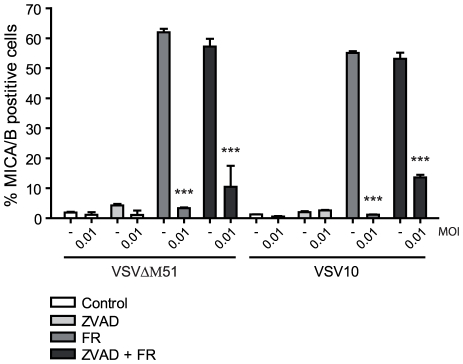
VSV-mediated down modulation of MICA/B surface expression is not caused by apoptosis. JE6-1 cells were incubated with 50 µM ZVAD-Fmk (ZVAD) just prior to mock infection (-) or infection with 0.01 MOI VSV10 or VSV^ΔM51^. One hr post infection, the cells were treated with 20 ng/mL FR901228 (FR), where indicated. 19 hr post infection, the cells were analyzed for MICA/B surface expression by flow cytometry. The bar graphs show mean±SD from three experiments. *** p<0.0001.

HDAC-inhibitors are known to augment VSV-induced apoptosis [27], so to exclude the possibility that the down modulation of FR901228-induced MICA/B expression was due to an increase in apoptosis by VSV, Jurkat T-cells were treated with the caspase inhibitor, ZVAD-Fmk. Treatment with ZVAD-Fmk abrogated the induction of apoptosis by VSV and VSV plus FR901228 (data not shown), which is consistent with previous findings [27]. The inhibition of apoptosis led to a minor increase in FR901228-induced MICA/B surface expression after VSV10 and VSV^ΔM51^ infection (Fig. 4), which indicates that apoptosis could be involved in the VSV blockade of HDAC-inhibitor mediated MICA/B expression. However, we did not observe any MICA/B surface expression on VSV infected cells, which were not treated with FR901228, after inhibition of apoptosis (Fig. 4). These data suggest that VSV must possess an additional ability to abrogate the surface expression of NKG2D-ligands. VSV infection caused a minor decrease in cell viability (<10%) after 19 hr as measured by PI exclusion (data not shown), suggesting that the down modulation of MICA/B is not caused by gross toxicity.

### VSV-mediated inhibition of MICA surface expression occurs at an early post transcriptional level

**Figure 5:**
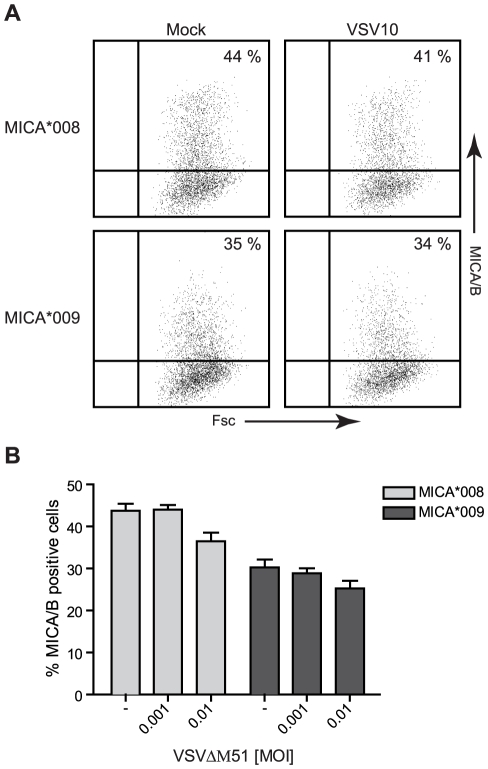
VSV-mediated inhibition of MICA surface expression occurs at an early post transcriptional level. ***A***, JTag-9 cells were transiently transfected with 5 µg MICA*008-GFP (MICA*008) or MICA*009-GFP (MICA*009) vector constructs. Two hr post transfection, the cells were either mock infected or infected with 0.001 MOI VSV10 for 19 hr. The cells were analyzed for surface MICA/B expression by flow cytometry. The dot-plots represent one out of three experiments. ***B***, JTag-9 cells were transiently transfected as described in *A*. Two hr post transfection, the cells were either mock infected (-) or infected with 0.001 or 0.01 MOI VSV^ΔM51^ for 19 hr. The cells were analyzed for MICA/B surface expression by flow cytometry. The bar graphs show mean±SD from three experiments.

HCMV can escape immune recognition by hindering surface expression of full-length MICA alleles, but not the truncated allele, MICA*008 [22]. To test whether VSV distinguish between different alleles of MICA during the down modulation, we transiently transfected Jurkat T-cells with vector constructs encoding the full-length MICA*009 allele and the truncated MICA*008 allele. As shown in Fig. 5A and B, neither VSV10 nor VSV^ΔM51^ abrogated the cmv-controlled surface expression of MICA*008 or MICA*009. VSV infection of the transfected cells caused cell death (VSV10) and GFP expression (VSV^ΔM51^), indicating that the transfection did not affect viral infection (data not shown).

The results suggest that VSV do not discriminate between the different alleles of MICA. Furthermore, the lack of inhibition of MICA after transient transfection indicates that VSV infection inhibits the cell surface expression of MICA at an early post transcriptional level. In agreement with this, we found that both VSV10 and VSV^ΔM51^ infection decrease the intracellular protein level of MICA/B after FR901228 treatment (Fig. 1C & 3B).

VSV is known to cause a global inhibition of translation during infection [30]–[32], however VSV infection did not affect MICA surface expression after transient over expression (Fig. 5A and 5B). cycloheximide (CHX), a potent inhibitor of translation, was used to verify that effective inhibition of translation could affect transient overexpression of MICA. Treatment with CHX 4 hr post transfection inhibited surface expression of MICA by 71.1%±1.8%, and treatment with CHX 12 hr post transfection inhibited surface expression of MICA by 34.1%±1.4%. Since transient over expression of MICA can be inhibited by CHX, but not by VSV infection, this implies that a global inhibition of translation is not the major course of VSV-mediated down modulation of MICA surface expression.

## Discussion

This report is the first showing an effect of VSV infection on NKG2D-ligand expression. We observed that infection of cancer cells with the oncolytic virus, VSV, leads to an induction of mRNA expression of the NKG2D-ligand, MICA. However, the increase in MICA mRNA expression was not followed by an increase in surface expression. VSV infection inhibited stress-induced and endogenous surface expression of several NKG2D-ligands, including MICA/B and ULBP-2, indicating that the VSV-mediated inhibition targets NKG2D-ligands from both the *MIC* and *RAET1* gene families. Furthermore, the VSV-mediated down modulation of NKG2D-ligand expression was observed in Jurkat T-cells and melanoma cells, showing that the down-modulation is not cell type specific.

VSV has an interest in keeping NKG2D-ligands away from the cell surface, especially since the virus infection leads to a robust induction of MICA mRNA. We did not discover the viral mechanism or gene product mediating the down modulation of NKG2D-ligands, and the precise molecular mechanism behind this remains to be identified. However, we did find that the well documented escape mechanism of VSV (i.e. inhibition of nucleocytoplasmic transport of mRNAs by the M protein [9]–[11]) was not involved in the down modulation of NKG2D-ligands on Jurkat T-cells. Thus, it appears that VSV possess an unknown escape mechanism targeting NKG2D-ligand mediated immune recognition.

The VSV M protein is involved in several stages of the infection [4]–[6], thus we cannot rule out an involvement of the M protein in the down modulation of NKG2D-ligand surface expression, by a mechanism distinct from nucleocytoplasmic transport.

We have previously shown that MICA/B is not up-regulated on apoptotic cells [24]. The down regulation of NKG2D-ligand expression could therefore be an outcome of VSV-induced apoptosis. Inhibition of apoptosis by treatment with ZVAD-Fmk only led to a minor increase in FR901228-induced MICA/B surface expression after VSV infection, and therefore apoptosis is likely not a key player in the down modulation of NKG2D-ligand expression by VSV.

NKG2D-ligand expression can be controlled by ubiquitinylation, and the E3 ubiquitin ligase K5 from Kaposai sarcoma virus has been shown to down regulate cell surface expression of MICA by ubiquitination of lysine residues in the protein's cytoplasmic tail [33]. Expression of the E3 ubiquitin ligase Triad3A is induced by VSV infection, and is an alternative way that VSV possess to inhibit the activation of an antiviral response [28]. Triad3A negatively regulates the RIG-I/MAVS signalling pathway through TRAF3 degradation [28], and promotes proteolytic degradation of some Toll-like receptors [34]. Triad3A could therefore be involved in the down modulation of NKG2D-ligands. We have tested the influence of Triad3A on NKG2D-ligand expression. However, overexpression of Triad3A in Jurkat T-cells weakly affected NKG2D-ligand expression following HDAC-inhibitor treatment and only at transfection levels that decreased cell viability (data not shown). We therefore find it less likely that Triad3A has a major role in the VSV-mediated down modulation of NKG2D-ligands.

A down modulation of FR901228-induced MICA/B cell surface expression was detectable 12 hr post VSV infection, which could imply that a certain level of VSV proteins must be present before the down modulation is observed. However, future studies needs to clarify if this effect is a direct or indirect result of the VSV infection.

Constitutive surface expression of MICA, following transient transfections with two different alleles of MICA (*009 & *008), could not be inhibited by VSV infection, thus the VSV-mediated block of NKG2D-ligand surface expression seems not to discriminate between the different alleles of MICA and to occur at an early post transcriptional level.

VSV infection is recognized for its global inhibition of host protein translation [30]–[32]. The MICA mRNA level is increased 4 hr after FR901228 treatment and 12 hr after VSV infection, it is therefore possible that the VSV-mediated reduction of MICA surface expression is caused by an inhibition of translation. In this study we examine MICA surface expression in three experimental setups: 1) after VSV infection; 2) after HDAC-inhibitor treatment +/− VSV infection and 3) after transient transfection with vector constructs encoding MICA +/− VSV infection. VSV infection inhibits MICA surface expression in 1) and 2), but not in 3), which suggests that global inhibition of translation is not the primary course of the VSV-mediated inhibition. It should be noted that 3) is a transient transfection with a MICA construct just prior to VSV infection, it is therefore unlikely that MICA is expressed if there is a global inhibition of translation. To examine this aspect further, we treated MICA-transiently transfected cells with CHX, a potent inhibitor of translation. Since treatment with CHX, and not VSV infection, led to a decrease in MICA surface expression, this suggests that a general inhibition of translation by VSV is not the primary mechanism of inhibition of MICA surface expression, at least not within the time frame and VSV concentrations used in our experimental settings.

Together our results suggest that VSV targets NKG2D-ligand surface expression by a mechanism different from other viruses. HCMV UL142 retains several MICA alleles (but not the truncated allele MICA*008) in the *cis*-golgi aparatus, but it does not affect MICB [18]; HCMV UL16 retain MICB, ULBP-1, and ULBP-2 in the ER [19]–[22]; HCMV micro-RNA specifically targets the 3′ untranslated region of the MICB transcript [35]; and adenovirus E3/19K retain both MICA (including MICA*008) and MICB in the ER, but does not affect ULBP-1–3 [23].

Previous efforts have shown that it is possible to modify the VSV genome to enhance the oncolytic properties of VSV, e.g. by adding cDNA encoding IL-4, which had a positive effect on VSV-induced oncolysis in experimental animal models [36]. Our results suggest that it may be beneficial to alter the VSV genome to accommodate surface expression of NKG2D-ligands, depending on the type of cancer. This will require a further delineation of the molecular mechanism behind VSV-mediated hindering of NKG2D-ligand surface expression, but will potentially boost the anti-tumor ability of VSV.

VSV is well-known for its oncolytic ability and the VSV-mediated regulation of NKG2D-ligands may have several effects in this regard.

Various tumors, particular Melanoma, Prostate, Ovarian and B-CLL cancers, are known to evade the immune system by shedding soluble NKG2D-ligands [37]–[40]. This shedding cause a persistent down modulation of NKG2D expression and leads to impaired anti-cancer activity by NK cells and CD8 T cells [41]. In this specific context, the VSV-mediated inhibition of NKG2D-ligand expression could have a favorable effect on immunity against cancer. Additional studies are however needed to address if VSV can inhibit the shedding of soluble NKG2D-ligands from cancer cells.

Interestingly, HDAC-inhibitors have been found to increase the oncolytic effect of VSV *in vivo* in different cancers [27]. In this particular circumstance, we would envisage that the synergistic induction of cancer cell death with VSV infection and HDAC-inhibitor treatment prevails over the lack of NKG2D-ligand expression It is however noteworthy that the study uses Melanoma and Prostate cancer cells, which may express suppressive soluble NKG2D-ligands.

In apparent contradiction to the results presented in the current manuscript, previous reports have shown that VSV infection results in a robust recruitment of NK and NK-T cells in immune-competent hosts [42], [43]. In our opinion the most likely explanation is that VSV infection induces other molecules that can recruit and activate NK cells. Although not limited to, likely candidates could be: Hsp70, DNAM-1, Calreticulin and HMGB1 [44]–[46]. Future studies need to clarify this potentially interesting effect of VSV infection on other immune stimulatory proteins.

The question is then why VSV inhibits NKG2D-ligand expression when other stimulatory molecules might be induced or not affected. Although speculative in nature there are several likely explanations: 1) NK cells have several stimulatory receptors e.g. NKG2D, DNAM-1, 2B4, NKp46 and others [47], and it has elegantly been shown that full NK cell activation is dependent upon engagement of several of these receptors in combination [48]. It is therefore possible that VSV blocks NKG2D engagement to prevent full NK cell activation, this may be particular important for VSV infection as it directly stimulates NK cell recruitment [42], [43]; 2) Diefenbach et al. have furthermore demonstrated that NKG2D-ligand expression by tumor cells is important for their ability to induce immunological memory [49]. Thus, it is possible that the NKG2D/NKG2D-ligand system plays a specific role in the development of a memory response against VSV.

The outcome of cancer treatment or co-treatment with VSV will depend on the relative contribution of different contradictory factors. Positive factors could be: the synergistic chemotherapeutic potential and the down modulation of tumor-mediated shedding of soluble NKG2D-ligands. Negative factors could be: the lack of induction of immunological memory due to reduced NKG2D-ligand expression and the lack of tumor recognition due to reduced NKG2D-ligand expression.

The focus of the current study has been to demonstrate and characterize the VSV-mediated suppression of MICA/B and ULBP2 surface expression. Since viruses rarely, if ever, target cellular proteins without a scope, we believe that our findings are a significant step forward in the understanding of VSV biology.
